# Corrigendum to “HD-13 Induces Swine Pneumonia Progression via Activation of TLR9”

**DOI:** 10.1155/2022/9841070

**Published:** 2022-11-16

**Authors:** Jing Liu, Jiayi Xu, Honglei Zhou, Lihua Wang

**Affiliations:** ^1^Jiangsu Agri-Animal Husbandry Vocational College, Taizhou, 225300 Jiangsu, China; ^2^Nanjing Agricultural University, Nanjing, 210095 Jiangsu, China

In the article titled “HD-13 Induces Swine Pneumonia Progression via Activation of TLR9” [1], there was an error in the Acknowledgments section. The corrected Acknowledgments section appears below:

This work was supported by grants from the Scientific Research Project of Jiangsu Agri-animal Husbandry Vocational College (NSF201903).

## References

[1] Liu J., Xu J., Zhou H., Wang L. (2022). HD-13 Induces Swine Pneumonia Progression via Activation of TLR9. *Computational and Mathematical Methods in Medicine*.

